# Regulatory Effects of Paclobutrazol and Uniconazole Mixture on the Morphology and Biomass Allocation of *Amorpha fruticosa* Seedlings

**DOI:** 10.3390/plants14233684

**Published:** 2025-12-03

**Authors:** Jiapeng Zhang, Ning Liu, Keyan Wu, Xueli Zhang, Chengcheng Gao, Fenfen Liu, Jimeng Sun, Chenggong Liu

**Affiliations:** 1 State Key Laboratory of Tree Genetics and Breeding, Research Institute of Forestry, Chinese Academy of Forestry, Beijing 100091, China; 18547607754@163.com (J.Z.); poplarning@caf.ac.cn (N.L.); zxl961109@163.com (X.Z.); gaocc@caf.ac.cn (C.G.); fen@caf.ac.cn (F.L.); sjm376131292@163.com (J.S.); 2 College of Desert Control Science and Engineering, Inner Mongolia Agricultural University, Hohhott 010010, China; 3 Baiyinhua Forest Farm, Horqin Left Middle Banner, Tongliao 028000, China; 18247802053@163.com; 4 Key Laboratory of Tree Breeding and Cultivation, State Forestry and Grassland Administration, Beijing 100091, China

**Keywords:** plant growth retardant, *Amorpha fruticosa*, biomass allocation, correlation analysis, comprehensive trait evaluation

## Abstract

Global climate change has intensified land desertification in the arid and semi-arid regions of northwestern China, highlighting the urgent need to cultivate plant species with ideal architecture and well-developed root systems to combat ecosystem degradation. *Amorpha fruticosa* is widely used as a windbreak and sand-fixation shrub; however, its rapid growth and high transpiration during the early planting stage often result in excessive water loss, low survival rates, and limited vegetation restoration effectiveness. Plant growth retardants (PGRts) are known to suppress apical dominance and promote branching. In this study, one-year-old *A. fruticosa* seedlings were treated with different combinations of paclobutrazol (PP_333_) and uniconazole (S_3307_) to investigate their effects on plant morphology and biomass allocation; it aims to determine the optimal formula for cultivating shrub structures with excellent windbreak and sand-fixation effects in land desertification areas. The results showed that both PP_333_ and S_3307_ significantly inhibited plant height while promoting basal stem diameter, branching, and root development. Among all treatments, the S_3307_ 200 mg·L^−1^ + PP_333_ 200 mg·L^−1^ combination (SD3) was the most effective, resulting in the greatest increases in basal diameter, branch number, total root length, and root-to-shoot ratio, while significantly reducing height increment, leaf length and leaf area (*p* < 0.05). Under the S_3307_ 200 mg·L^−1^ + PP_333_ 300 mg·L^−1^ treatment (SD4), leaf width and specific leaf area were reduced by 17.92% and 38.89%, respectively, compared with the control. Correlation analysis revealed significant positive or negative relationships among most growth traits, with leaf length negatively correlated with other morphological indicators. Fresh and dry weights of both aboveground and root tissues were significantly positively correlated with basal diameter (*R* = 0.38) and branch basal diameter (*R* = 0.33). Principal component analysis demonstrated that the SD3 treatment achieved the highest comprehensive score (2.91), indicating its superiority in promoting a compact yet robust plant architecture. Overall, the SD3 treatment improved drought resistance and sand-fixation capacity of *A. fruticosa* by “dwarfing and strengthening plants while optimizing root–shoot allocation.” These findings provide theoretical support for large-scale cultivation and vegetation restoration in arid and semi-arid regions and offer a technical reference for growth regulation and windbreak and sand-fixation capacity in other xerophytic shrub species.

## 1. Introduction

Land desertification poses one of the most severe global environmental challenges, jeopardizing ecological security and sustainable development [1,2,3]. Under the accelerating trend of global warming, the frequency and intensity of extreme climate events such as droughts, heatwaves, and wildfires have increased, resulting in the rapid expansion and exacerbation of land degradation in arid and semi-arid regions [4,5]. It is estimated that nearly 75% of the Earth’s terrestrial ecosystems are currently experiencing varying levels of degradation, impacting over two billion people and threatening food security, biodiversity, and ecosystem stability at a global scale [6,7]. China is one of the countries most seriously affected by desertification, particularly in its northwestern regions such as the Mu Us Desert, Horqin Sandy Land, and Ulan Buh Desert, where desertified land accounts for approximately 27% of the national territory [8,9]. These regions are characterized by harsh climatic conditions and fragile ecosystems, lending to significant vegetation loss, soil degradation, and declining productivity, which underline the urgent need for effective ecological restoration strategies [9,10,11,12,13].

Among the various measures for desertification control, phytodesertification control occupies an important position due to its unique advantages, yet it also faces practical challenges. Vegetation plays a critical role in combating desertification by reducing wind speed, stabilizing dunes, trapping mobile sand particles, and improving soil conditions [14,15]. The aboveground plant structure (including leaves, branches, stems, and canopy) and the belowground root system jointly determine the effectiveness of windbreak and sand-fixation functions [16,17]. Studies have demonstrated that factors such as vegetation height, coverage, configuration, branch flexibility, and spatial distribution significantly influence wind speed and airflow, thereby affecting the windbreak function of plants [18,19]. Among these, plants with higher branching rates, larger branching angles [20], and dwarf tree [21] exhibit better windbreak and sand-fixation performance. Although vegetation restoration in desertified areas provides significant ecological, economic, and societal benefits, contributing to long-term environmental sustainability [22]. However, the success of such restoration efforts largely hinges on the cultivation and domestication of plant species possessing appropriate structural characteristics and root system morphologies, as environmental conditions such as drought, high temperatures, strong winds, and low soil fertility frequently often limit plant establishment and survival in these regions [9,23]. Therefore, optimizing plant architecture through targeted regulation represents a critical approach to improving the adaptability and ecological performance of vegetation in desertification control programs.

To address the bottlenecks in phytodesertification control, the characteristics and applications of plant growth retardants offer a novel approach for us. Plant growth retardants (PGRts) are a class of plant growth regulators that inhibit endogenous gibberellin biosynthesis, thereby suppressing apical dominance, reducing stem elongation, and promoting lateral branching [24,25]. The application of PGRts has been widely documented in agricultural crops, fruit trees, and ornamental horticulture [25,26,27,28], where they have been shown to reduce plant height, enhance stem diameter, increase chlorophyll content, and improve stress tolerance [29,30,31,32,33]. Among the commonly used growth retardants, paclobutrazol (PP_333_) primarily inhibits gibberellin synthesis, lending to reduced internode elongation and suppressed shoot growth [34]. In contrast, uniconazole (S_3307_) inhibits abscisic acid catabolism, enhances photosynthetic capacity, and improves antioxidant activity [35,36,37]. Previous studies have demonstrated that PP_333_ effectively reduces plant height in rice and pomegranate [25,38], while S_3307_ promotes root development in wheat and increases stem diameter in japonica rice [39,40]. However, despite these promising effects, the application of these growth retardants in forestry species, particularly in xerophytic shrubs utilized for larges-cale vegetation restoration in arid and semi-arid regions, remains underexplored.

*A. fruticosa* is a perennial, multi-stemmed shrub of the family Leguminosae that is widely used in northwestern China for windbreak, sand fixation, and ecological restoration [41]. This species is characterized by its extensive lateral root system, strong regenerative capacity, and high adaptability to poor soils, enabling it to effectively reduce wind erosion and stabilize sandy surfaces [42]. However, during the initial establishment stage, *A. fruticosa* exhibits rapid vegetative growth and high transpiration rates, which can lead to excessive water consumption and reduce survival under arid conditions [43]. These physiological characteristics, combined with severe environmental stressors, frequently result in low establishment rates and hinder its large-scale application in desertification control. As such, regulating the architectural traits of *A. fruticosa* to improve its root-to-shoot allocation and stress resistance is crucial for improving its ecological effectiveness in arid regions.

In this study, *A. fruticosa* seedlings were treated with different combinations of paclobutrazol (PP_333_) and uniconazole (S_3307_) to evaluate their effects on plant morphology, root development, and biomass allocation. A principal component analysis (PCA) method was employed to assess the comprehensive effects of these treatments. The objectives of this study were to: (i) elucidate the regulatory effects of different PP_333_ and S_3307_ formulations on phenotypic traits and biomass allocation in *A. fruticosa* seedlings; (ii) clarify the correlations among key growth traits through correlation analysis; and (iii) identify the optimal growth retardant combination for achieving dwarfing with enhanced robustness and improved biomass allocation efficiency using principal component analysis. The findings of this study are expected to provide a theoretical basis for the rational application of plant growth retardants in xerophytic shrubs and it provides technical support and theoretical reference for the plant type regulation, largescale cultivation, and vegetation restoration of desert plants represented by *A. fruticosa*.

## 2. Results

### 2.1. Effects of Plant Growth Retardants on Seedling Height and Basal Stem Diameter

It was found through studying the effects of PP_333_ and S_3307_ on the growth dynamics of plant height and basal diameter of *A. fruticosa* seedlings (Table 1) that, when the treatment duration reached 15 days, seedlings exhibited the fastest height increment, yet all treatments significantly reduced height growth relative to CK (*p* < 0.05). As treatment duration progressed, between-group differences gradually narrowed. By 90 days, SD3 (200 mg·L^−1^ S_3307_ + 200 mg·L^−1^ PP_333_) produced the lowest height increment (7.40 cm), only 11.35% of CK, indicating a strong dwarfing effect. In contrast, all treatments increased basal diameter (BDH), with the strongest promotion occurring early after application; the effect weakened over time. SD3 yielded the greatest BDH increment (3.45 mm), 4.49-fold that of CK, demonstrating that appropriate PP_333_–S_3307_ combinations can suppress excessive height while enhancing stem thickening and architectural stability.

**Figure 1:**
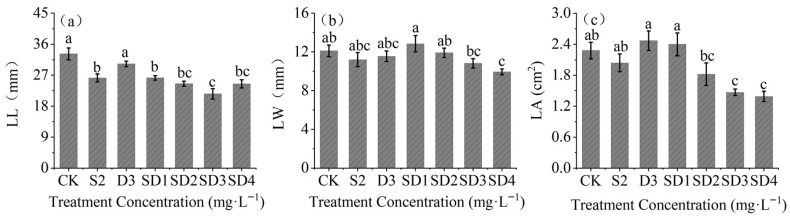
Effects of mixed agents of PP_333_ and S_3307_ on leaf traits of *A. fruticosa* seedlings. (**a**) leaf length (LL); (**b**) leaf width (LW); (**c**) leaf area (LA). Different lowercase letters indicate significant differences at the 0.05 level.

Analysis of leaf traits (Figure 1) revealed that, except for the D3 treatment significantly decreased LL compared with CK (*p* < 0.05). SD3 produced the smallest LL (21.57 mm), a 26.30% reduction versus CK (*p* < 0.05). LW reached its minimum under SD4 (9.94 mm), 17.92% lower than CK, whereas other treatments showed no significant LW change. LA was significantly reduced by SD3 and SD4 (*p* < 0.05), by 35.53% and 38.89% relative to CK, respectively; differences for other treatments were not significant (*p* > 0.05).

### 2.2. Effects of Plant Growth Retardants on Root System Development

**Figure 2:**
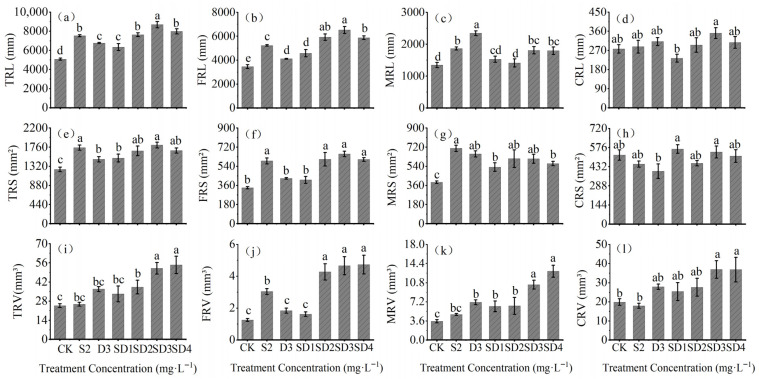
Effects of mixed agents of PP_333_ and S_3307_ on root growth of *A*. *fruticosa* seedlings. (**a**) Total root length (TRL), (**b**) Fine root length (FRL), (**c**) Medium root length (MRL), (**d**) Coarse root length (CRL), (**e**) Total root surface area (TRS), (**f**) Fine root surface area (FRS), (**g**) Medium root surface area (MRS), (**h**) Coarse root surface area (CRS), (**i**) Total root volume (TRV), (**j**) Fine root volume (FRV), (**k**) Medium root volume (MRV), and (**l**) Coarse root volume (CRV). Different lowercase letters indicate significant differences at the 0.05 level.

All treatments significantly increased TRL and TRS compared with the control (*p* < 0.05) (Figure 2a,b). Among them, the SD3 treatment resulted in the greatest enhancement of TRL, reaching 1.71 times that of the CK, while the S2 treatment exhibited the highest TRS, which was 1.60 times that of the CK. Furthermore, the SD3 treatment markedly promoted FRL and FRS, increasing them by 88.89% and 93.86%, respectively, compared with CK (Figure 2c,d).

The MRL and MRS were observed under the D3 and S2 treatments, with values of 233.95 mm and 709.3 mm^2^, respectively (Figure 2e,f). In contrast, no significant effects on CRL and CRS were detected across treatments (Figure 2g,h). Additionally, as shown in Figure 2i–l, the SD4 treatment most effectively stimulated TRV, FRV, and MRV, which increased to 2.21, 3.75, and 3.66 times those of CK, respectively. CRV also increased significantly under SD3 and SD4 treatments, by 85.95% and 85.50%, respectively (*p* < 0.05).

According to Table 2, fine roots accounted for the largest proportion of both root fresh weight (RFW) and root dry weight (RDW), exceeding 40%. The proportions of root surface area (RS) among fine, medium, and coarse roots were relatively balanced, ranging from 20% to 40%. In contrast, coarse roots contributed the highest proportion to total root volume (RV), accounting for more than 60%.

### 2.3. Effects of Plant Growth Retardants on Biomass Allocation of Seedlings

**Figure 3:**
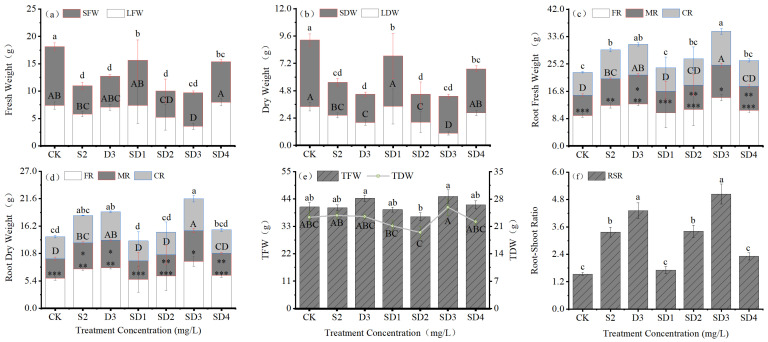
Effects of mixed agents of PP_333_ and S_3307_ on biomass allocation in *A. fruticosa* seedlings. (**a**) leaf fresh weight (LFW) and stem fresh weight (SFW), (**b**) leaf dry weight (LDW) and stem dry weight (SDW), (**c**) root fresh weight, (**d**) root dry weight (FR: fine roots, MR: medium roots, CR: coarse roots), (**e**) total fresh weight (TFW) and total dry weight (TDW), (**f**) root-to-shoot ratio (RSR). Different lowercase and uppercase letters. indicate significant differences at the 0.05 level. The asterisks (*, **, and ***) denote the significance of differences between various treatments for the same trait at the 0.05 level in the figure (i.e., an identical number of asterisks within the same layer signifies no significant difference (*p* > 0.05), whereas varying numbers of asterisks indicate a statistically significant difference (*p* < 0.05)).

Analysis of plant biomass showed that, the application of growth retardants reduced the leaf fresh weight (LFW), leaf dry weight (LDW), stem fresh weight (SFW), and stem dry weight (SDW) of *A*. *fruticosa* seedlings. Under the SD3 treatment, LFW and LDW were the lowest, reaching 0.48 and 0.31 times those of CK, respectively. SFW and SDW were the lowest under SD2, decreasing by 55.29% and 58.26% compared with CK (Figure 3a,b).

Figure 3c,d show that root fresh weight and root dry weight of fine, medium, and coarse roots increased in all treatments. The SD3 treatment exhibited the highest values, which were significantly higher than CK (*p* < 0.05), reaching 1.59-, 1.58-, and 1.49-fold increases in root fresh weight and 1.57-, 1.57-, and 1.45-fold increases in root dry weight for fine, medium, and coarse roots, respectively.

As shown in Figure 3e, there were no significant differences among treatments in total fresh weight (TFW) and total dry weight (TDW). Figure 3f indicates that the root–shoot ratio (RSR) increased significantly in all treatments except SD1 and SD4 (*p* < 0.05), with the highest value observed under SD3, reaching 5.04, 228.54% higher than CK.

### 2.4. Effects of Plant Growth Retardants on Branch Growth

**Figure 4:**
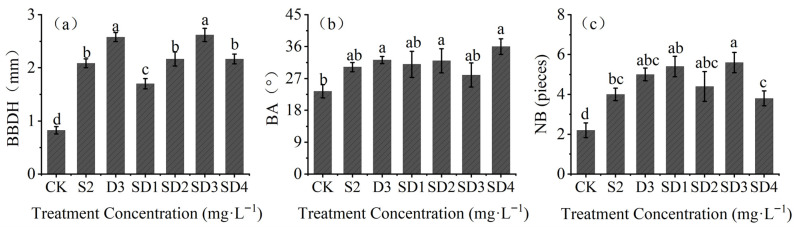
Effects of mixed agents of PP_333_ and S_3307_ on branch basal diameter, branch angle, and number of branches in *A*. *fruticosa* seedlings. (**a**) branch basal diameter (BBDH); (**b**) branch angle (BA); (**c**) number of branches (NB). Different lowercase letters indicate significant differences at the 0.05 level.

Studies on branching characteristics showed that, the branch basal diameter (BBDH) of seedlings in all treatments was significantly higher than that of CK (*p* < 0.05). The SD3 treatment showed the highest BBDH, which was 3.16 times that of CK (Figure 4a). Figure 4b indicates that branch angle (BA) under the S2, SD1, and SD3 treatments did not differ significantly from CK, whereas other treatments resulted in significantly larger BA values (*p* < 0.05). The largest branch angle was observed in the SD4 treatment (36.0°), 53.85% higher than CK. As shown in Figure 4c, all treatments significantly increased branch number (NB) compared with CK, with the SD3 treatment showing the highest NB (5.6), 1.55 times that of CK.

### 2.5. Correlation Analysis of Morphological Traits Under Different Treatments

**Figure 5:**
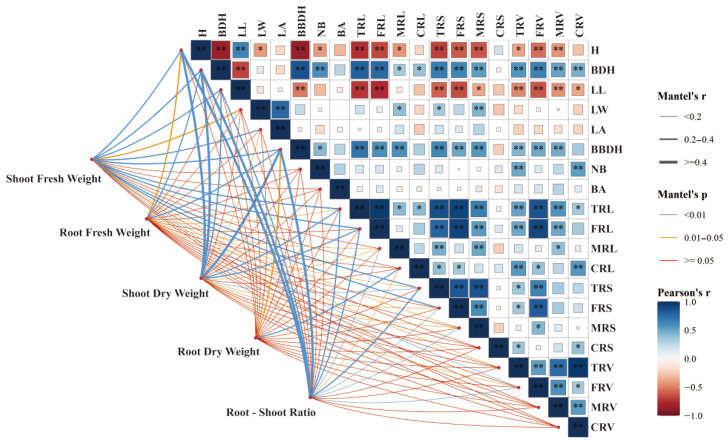
Correlation analysis of morphological traits of *A*. *fruticosa* seedlings under mixed agents of PP_333_ and S_3307_ treatments. * indicates a significant correlation at *p* < 0.05, ** indicates an extremely significant correlation at *p* < 0.01.

Correlation analysis indicated that (Figure 5), varying degrees of correlation were observed among the growth traits of *A. fruticosa* seedlings under different combinations of plant growth retardants. Plant H was positively correlated with LL (*R* = 0.64) and negatively correlated with BBDH (*R* = −0.85). Basal diameter (BDH) was negatively correlated with LL (*R* = −0.68) and positively correlated with BBDH (*R* = 0.85). LL showed a significant negative correlation with FRL (*R* = −0.76), while LW was strongly positively correlated with LA (*R* = 0.77).

BBDH was positively correlated with TRL (*R* = 0.70), and NB exhibited a moderate positive correlation with CRV (*R* = 0.52). TRL was very strongly correlated with FRL (*R* = 0.94), and FRL was strongly correlated with FRS (*R* = 0.91). MRL and MRS were positively correlated (*R* = 0.50), and CRL was positively correlated with CRV (*R* = 0.56). TRS was positively correlated with MRS (*R* = 0.86), and FRS was strongly correlated with FRV (*R* = 0.82). MRS and FRV showed a moderate positive correlation (*R* = 0.41), and TRV was very strongly correlated with CRV (*R* = 0.96). FRV was correlated with MRV (*R* = 0.58), and MRV was correlated with CRV (*R* = 0.53).

From a biomass perspective, aboveground fresh weight showed a positive correlation with overall growth traits, particularly with BDH (*R* = 0.38). Root fresh weight (RFW) exhibited weak positive correlations with H, BDH, and BBDH, with the highest correlation observed with BBDH (*R* = 0.33). Aboveground dry weight and RSR were positively correlated with H (*R* = 0.40, 0.41), BDH (*R* = 0.44, 0.43), and BBDH (*R* = 0.48, 0.59), respectively. RDW showed weak positive correlations with BBDH (*R* = 0.23) and CRL (*R* = 0.29).

### 2.6. Principal Component Analysis and Comprehensive Evaluation

Based on principal component analysis (PCA) of 33 phenotypic traits of *A. fruticosa* seedlings (Table 3), seven principal components were extracted, with a cumulative contribution rate of 86.48%. The first principal component (PC1) included FRFW, MRFW, CRFW, FRDW, MRDW, CRDW, TFW, TDW, and RSR, with a contribution rate of 41.01%. The second principal component (PC2) included TRL, FRL, TRS, FRS, MRS, and FRV, with a contribution rate of 15.35%. The third principal component (PC3) included H, BBDH, SFW, LDW, SDW, and RSR, accounting for 10.22% of the total variance. The fourth principal component (PC4) comprised LW and LA, contributing 6.96%. The fifth principal component (PC5) included NB and BA, contributing 6.07%. The sixth principal component (PC6) was composed of TRV, MRV, and CRV, with a contribution rate of 3.83%. The seventh principal component (PC7) included CRS, accounting for 3.04% of the total variance.

Comprehensive evaluation based on principal component scores (Table 4) showed that the SD3 treatment had the highest comprehensive score (2.91), ranking first among all treatments. SD4 ranked second, followed by D3 and SD2, while CK had the lowest comprehensive score.

## 3. Discussion

Vegetation-based sand control has been widely recognized as one of the most effective strategies for mitigating land desertification, particularly in arid and semi-arid regions where wind erosion is severe [44]. Plant architecture is a direct manifestation of plant environmental adaptability and plays a crucial role in determining ecological functions such as wind resistance, soil stabilization, and water conservation [45,46,47]. However, plant morphological traits are dynamic and can be significantly modified by both environmental factors and growth regulatory substances [48]. In this study, all mixed applications of paclobutrazol and uniconazole inhibited plant height and leaf length while promoting basal diameter, branch basal diameter, number of branches, and branch angle in *A*. *fruticosa* seedlings. The SD3 treatment demonstrated the most potent inhibitory effect on plant height and the most significant improvements in basal diameter and branch number, indicating that this combination effectively promotes a more compact and structurally stable architecture. The SD4 treatment induced the greatest increase in branch angle, suggesting a favorable modification for enhancing canopy spatial distribution. Both treatments also significantly suppressed leaf growth, likely due to the ability of paclobutrazol and uniconazole to inhibit gibberellin biosynthesis and reduce cell elongation, resulting in morphological dwarfing [34]. Moreover, these growth retardants promote cell division and increase the number of cell layers, thereby enhancing stem thickening and structural robustness [42], consistent with previous findings by Shahzad et al. [49], Lin et al. [50], and Ebmeyer et al. [51].

Roots are essential organs for water and nutrient uptake and play a critical role in plant survival, particularly during the early growth stages in arid environments [52]. Fine roots primarily function in resource absorption, while coarse roots are responsible for transport and mechanical support [53,54]. Root traits differ significantly among vegetation types and strongly influence ecosystem processes such as hydrological regulation, nutrient cycling, and resistance to wind erosion [55,56,57,58]. In this study, all PP_333_ and S_3307_ combinations significantly promoted the root development of *A. fruticosa*. The SD3 treatment resulted in the greatest increases in total root length, fine root length, fine root surface area, and coarse root volume, whereas the SD4 treatment yielded the highest total root volume and volumes of fine, medium, and coarse roots. These results suggest that combined treatments are more effective than single-agent applications, indicating a potential additive or synergistic effect. Fine roots accounted for the largest proportion of root fresh and dry weights across all treatments, indicating that growth retardants primarily enhanced absorptive root development. These responses may be attributed to the inhibitory effects of paclobutrazol and uniconazole on primary root elongation and cell expansion, coupled with their stimulatory role in lateral root proliferation and cell layer formation. Consequently, the increased fine root development likely contributes to enhanced water acquisition and improved tolerance to environmental stress. These findings align with previous studies reporting that plant growth retardants enhance root system architecture [59,60,61,62].

Biomass allocation is a key adaptive strategy by which plants respond to environmental conditions, optimizing growth and resource use efficiency [63,64,65]. In desert ecosystems, plants typically allocate more biomass to belowground organs to enhance water uptake and improve survival under drought and nutrient-poor conditions [66]. Watson et al. [67] reported that root biomass increases when shoot growth is restricted and photosynthetic assimilates are preferentially allocated to belowground tissues. In this study, all retardant treatments decreased leaf and stem fresh and dry weights while significantly increasing root biomass, leading to higher root–shoot ratios, particularly under the SD3 treatment. This indicates that growth retardants facilitated a shift in biomass allocation patterns, favoring root development over shoot growth. Although total biomass showed no significant differences among treatments, the redistribution of biomass towards root tissues suggests an adaptive response that enhances windbreak and sand-fixation capacity in arid environments. These findings are consistent with Sara et al. [68] who reported similar shifts in biomass allocation under growth retardant treatments in xerophytic species.

Comprehensive analysis of plant architecture, root development, and biomass allocation revealed that the mixed application of PP_333_ and S_3307_ significantly altered the morphological structure and physiological responses of *A. fruticosa* seedlings. Although growth retardants reduced plant height, total biomass exhibited an increasing trend under combined treatments, suggesting that the growth-promoting effects of mixed applications outweigh the inhibitory effects observed in single treatments. Morphological modifications such as reduced height and increased basal diameter contribute to a lower center of gravity, thereby decreasing lodging risk and enhancing structural stability. Furthermore, The reduction in plant height, thickening of basal diameter and branch basal diameter, increase in branch number, and enlargement of branch angle of *A*. *fruticosa* can not only protect the ground surface but also better intercept wind and sand. The enhanced root system further improves lodging resistance and increases soil water absorption, ultimately contributing to enhanced windbreak and sand-fixation capabilities. Song et al. [43] showed that 300 mg·L^−1^ paclobutrazol had the best regulatory effect on *A*. *fruticosa* seedlings; Zhang et al. [69] reported that among single-agent treatments, 300 mg·L^−1^ paclobutrazol and 200 mg·L^−1^ uniconazole exhibited the optimal regulatory effects on *A*. *fruticosa* seedlings. These findings are inconsistent with the results of our study, which indicated that the mixed-agent treatment SD3 had a better regulatory effect on *A*. *fruticosa* seedlings than the two optimal single-agent concentration treatments. This may be attributed to the different mechanisms of action between paclobutrazol and uniconazole [34,35,36], and their combination produces a certain synergistic effect, which is consistent with the research results of Qian et al. [70]. Our study demonstrated that the regulatory effect of SD3 treatment on *A*. *fruticosa* seedlings was the best, superior to that of the mixed agent composed of the two optimal single-agent concentrations. This could be because both paclobutrazol and uniconazole are gibberellin-antagonistic growth inhibitors. Mixing their optimal concentrations may cause phytotoxicity in plants: high concentrations might excessively inhibit gibberellin synthesis in plants, thereby arresting plant growth and inducing phytotoxic traits [25]. However, influenced by plant growth stages and environmental factors, further verification and con-sideration are required regarding the selection of appropriate growth retardants and dos-ages, as well as whether these findings are universally applicable to most plants dependent on arid regions for survival.

## 4. Materials and Methods

### 4.1. Plant Materials

In April 2019, current-year cuttings (12 cm in length and 1.50 mm in diameter) with uniform size were collected from six-year-old *A*. *fruticosa* mother plants. The cuttings were inserted into seedling pots (21.80 cm in diameter and 21.70 cm in height) in a greenhouse at Inner Mongolia Agricultural University (111.717926° E, 40.810654° N), with one seedling per pot and a total of 140 seedlings. The relative humidity inside the greenhouse ranged from 35% to 70%, with an average temperature of 22 °C. Before watering, the initial total weight of each pot including substrate and container was 6.87 kg. The substrate consisted of field soil and nutrient soil at a volume ratio of 4:1, with a pH of 6.62, a volumetric water content of approximately 45.97%, and a maximum water-holding capacity of 64.27%. During the nursery period, seedlings were irrigated with 700 mL of water every 3 days. The position of each pot was rotated diagonally every 7 days, and weed removal, pest control, and soil loosening were conducted every 10 days to ensure uniform environmental conditions and avoid the effects of light, moisture, temperature, and pest damage on seedling growth.

### 4.2. Experimental Design

At the end of April 2019, when the lower part of the cuttings had reached a semi-lignified stage, 35 pots of *A. fruticosa* seedlings with similar growth status and vigor were selected to minimize individual variation and ensure the reliability of subsequent experiments. Prior to treatment, plants were watered to full saturation, and after free drainage of excess soil water from the pots, treatments were applied according to the method of Chen [71]. Each seedling was subjected to paclobutrazol (PP_333_, 15% wettable powder, Sichuan Guoguang Agrochemical Co., Ltd., Chengdu, China) or uniconazole (S_3307_, 5% wettable powder, Sichuan Guoguang Agrochemical Co., Ltd., Chengdu, China), or their combinations. Treatments were applied via soil drench at 16:00 on a clear afternoon (30 April 2019). Based on previous studies [69], Paclobutrazol (200 mg·L^–1^, 300 mg·L^–1^) and Uniconazole (100 mg·L^–1^, 200 mg·L^–1^) were selected, along with the concentration that shows the best regulatory effect on *A. fruticosa*. A total of six retardant treatments (S2, D3, SD1, SD2, SD3, and SD4) and one control (CK) were included. The application concentrations, dosages, and number of replicates are shown in Table 5. To prevent loss of applied solution, mats were placed under each pot, and 700 mL of water was applied every 3 days; leachate was collected and returned to the corresponding pots on a regular basis.

### 4.3. Measurement of Growth Parameters

#### 4.3.1. Plant Height and Basal Diameter

Before treatment (30 April 2019), plant height and basal diameter of each seedling were measured using a steel tape (YM-CL001, Yuma Tools Co., Ltd., Zhengzhou, China) and a vernier caliper (Diangjiang Technology Co., Ltd., Shanghai, China), respectively, as the initial values. After treatment, five seedlings from each treatment were randomly selected every 15 days, and plant height and basal diameter were measured to calculate the increments.

#### 4.3.2. Branch and Leaf Traits

After 90 days of treatment, branch number and branch basal diameter of five seedlings in each treatment were recorded, and the branch angle of the primary branches was measured using a protractor (Starrett, L.S. Starrett Company, Athol, MA, USA). Three fully expanded functional leaves from each seedling were selected (15 functional leaves were measured per treatment), and leaf length, leaf width, and leaf area were measured using a leaf area meter (Yaxin-1242, Beijing Yaxin Liyi Technology Co., Ltd., Beijing, China).

#### 4.3.3. Root Trait Measurement

At the end of the experiment, the soil in each pot was gently washed off with tap water. The root system was separated from the shoot and cleaned to remove any remaining debris. Leaves and stems were separated from the aboveground parts, and roots were classified into fine roots (<1.00 mm), medium roots (1.00–2.00 mm), and coarse roots (>2.00 mm) according to root classification standards. Each root class was placed in a labeled paper envelope, and fresh weight was recorded, five replicates per treatment. The root samples were then scanned using an Epson Expression 10000XL scanner (Epson, Long Beach, CA, USA), and root morphological parameters, including root length, surface area, and volume, were analyzed using WinRhizo software (v.2013e).

#### 4.3.4. Biomass Measurement

Biomass was determined using the oven-drying method. After measuring the fresh weight, the various organs (leaves, stems, and different root classes) were placed in an oven, heated at 105 °C for 0.5 h for enzyme deactivation, and then dried at 75 °C to a constant weight. Dry weights were recorded, and the root-to-shoot ratio (RSR) was calculated.

### 4.4. Data Processing and Analysis

Data organization and preprocessing were conducted using Excel 2023. Descriptive statistics, one-way analysis of variance (ANOVA), and Duncan’s multiple range test were performed using SPSS software (version 21.0). Growth curve fitting was carried out using Forstat software (version 2.2) [37]. Figures, tables, and correlation analyses were generated using Excel 2023, Origin 2023, and R software (version 3.1.2).

A membership function method was employed to normalize the data, and principal component analysis (PCA) was used to comprehensively evaluate the growth performance of *A. fruticosa* seedlings under different plant growth retardant treatments. In principal component analysis, the degree of association between an indicator and a principal component is mainly determined by the absolute value of the factor loading. It is generally considered that the closer the absolute value of the factor loading is to 1, the stronger the association between the indicator and that principal component [72,73]. The membership function was calculated using the following formulas [74]:

(1) $$x\;(\mu )\;=\;(x\;-{\;x}_{\min})/(x_{\max}\;-\;x_{\min})$$

(2) $$x\;(\mu )=1-\;(x-{\;x}_{\min})/(x_{\max}-x_{\min})$$

where x (μ) is the standardized value of the corresponding index, $x_{\max}$ is the maximum value of that index across all treatments, and $x_{\min}$ is the minimum value. Formula (1) was used for indicators positively correlated with treatment effects, while Formula (2) was applied for negatively correlated indicators.

## 5. Conclusions

Land desertification is one of the major challenges to global ecosystem stability and human survival. Exploring methods that promote plant growth and structural modification in the region is a critical task for enhancing vegetation for sand fixation and mitigating the pace of desertification. In this study, the perennial shrub *A. fruticosa*, which is commonly distributed in arid and semi-arid sandy regions, was used to investigate the effects of different combinations of two plant growth retardants, paclobutrazol (PP_333_) and uniconazole (S_3307_), on seedling growth characteristics and biomass allocation. The results showed that the mixed application of PP_333_ and S_3307_ effectively regulated the growth of *A. fruticosa* seedlings, enhanced stress resistance, and improved early survival. Among all treatments, the combination of 200 mg·L^−1^ S_3307_ + 200 mg·L^−1^ PP_333_ exhibited the best improvement in plant architecture, reducing height increment to 11.35% of the control while increasing basal diameter and branch number to 4.49-fold and 1.55-fold of the control, respectively. Leaf length and area were significantly reduced, indicating an adaptive morphological response to the treatment. Correlation analysis further revealed a strong positive relationship between root development and aboveground growth, highlighting the critical role of root systems in overall plant performance. However, the study was limited to one-year-old seedlings, and future research should consider different growth stages and environmental conditions, as well as evaluate the applicability of this retardant combination to other xerophytic shrub species to broaden its use in windbreak and sand-fixation practices.

## Figures and Tables

**Table 1:** Effects of mixed agents of PP_333_ and S_3307_ plant growth retardants on the growth of *A. fruticosa* seedlings. H: height increment; BDH: basal diameter increment. Different letters in the same column indicate significant differences at the 0.05 level.

Index	Treatment Concentration (mg·L^–1^)	Treatment Time (d)					
		15	30	45	60	75	90
H (cm)	CK	22.24 ± 0.49 a	17.60 ± 1.00 a	8.62 ± 0.46 a	1.88 ± 0.14 a	0.86 ± 0.08 a	0.20 ± 0.04 ab
	S2	4.36 ± 0.16 cd	2.86 ± 0.18 bc	0.52 ± 0.04 d	0.20 ± 0.03 e	0.10 ± 0.03 c	0.08 ± 0.04 b
	D3	5.20 ± 0.52 c	3.86 ± 0.31 bc	2.40 ± 0.20 b	1.44 ± 0.25 b	0.84 ± 0.17 a	0.20 ± 0.06 ab
	SD1	7.74 ± 0.44 b	4.10 ± 0.26 b	2.28 ± 0.27 b	1.04 ± 0.16 c	0.50 ± 0.05 b	0.14 ± 0.05 ab
	SD2	7.11 ± 0.51 b	3.24 ± 0.32 bc	1.52 ± 0.14 c	0.72 ± 0.12 cd	0.30 ± 0.05 bc	0.14 ± 0.04 ab
	SD3	3.76 ± 0.25 d	1.44 ± 0.09 d	1.02 ± 0.04 cd	0.54 ± 0.05 de	0.40 ± 0.07 b	0.28 ± 0.06 a
	SD4	5.62 ± 0.62 c	2.56 ± 0.40 cd	1.34 ± 0.22 c	0.72 ± 0.07 cd	0.28 ± 0.04 bc	0.26 ± 0.05 a
BDH (mm)	CK	0.28 ± 0.02 d	0.19 ± 0.02 d	0.13 ± 0.02 c	0.09 ± 0.009 c	0.05 ± 0.007 b	0.02 ± 0.006 a
	S2	0.89 ± 0.05 c	0.74 ± 0.04 ab	0.44 ± 0.05 ab	0.17 ± 0.03 ab	0.07 ± 0.03 ab	0.02 ± 0.006 a
	D3	0.93 ± 0.06 c	0.74 ± 0.05 ab	0.51 ± 0.05 a	0.19 ± 0.03 ab	0.08 ± 0.005 ab	0.04 ± 0.02 a
	SD1	0.92 ± 0.05 c	0.58 ± 0.05 c	0.36 ± 0.03 b	0.14 ± 0.01 bc	0.11 ± 0.03 a	0.03 ± 0.01 a
	SD2	1.31 ± 0.05 b	0.64 ± 0.06 bc	0.43 ± 0.05 ab	0.19 ± 0.02 ab	0.11 ± 0.02 a	0.02 ± 0.009 a
	SD3	1.78 ± 0.06 a	0.80 ± 0.04 a	0.52 ± 0.05 a	0.23 ± 0.03 a	0.09 ± 0.009 ab	0.01 ± 0.007 a
	SD4	0.93 ± 0.03 c	0.73 ± 0.02 ab	0.42 ± 0.05 ab	0.23 ± 0.01 a	0.11 ± 0.01 a	0.03 ± 0.007 a

**Table 2:** The Proportion of each root class in total root system of *A*. *fruticosa* under the application of a mixed agent of PP_333_ and S_3307_ (%). RFW: root fresh weight, RDW: root dry weight, RS: root surface area, RV: root volume, FR: fine roots, MR: medium roots, CR: coarse roots, same below. Different lowercase letters in the same row indicate significant differences at the 0.05 level.

Traits	Root System Classification	CK	S2	D3	SD1	SD2	SD3	SD4
RFW	FR	41.25 ± 0.58 a	42.11 ± 0.55 a	41.35 ± 0.60 a	42.05 ± 0.55 a	41.84 ± 0.49 a	42.17 ± 0.58 a	41.49 ± 0.60 a
	MR	27.66 ± 0.68 a	27.89 ± 0.55 a	28.43 ± 0.68 a	27.97 ± 0.54 a	27.80 ± 0.58 a	28.13 ± 0.49 a	28.29 ± 0.67 a
	CR	31.09 ± 0.80 a	30.00 ± 0.44 ab	30.22 ± 0.37 ab	29.97 ± 0.45 ab	30.36 ± 0.40 ab	29.70 ± 0.40 ab	30.22 ± 0.37 b
RDW	FR	41.79 ± 1.07 a	42.21 ± 0.86 a	41.82 ± 0.80 a	42.78 ± 0.86 a	42.40 ± 0.40 a	42.79 ± 1.03 a	41.86 ± 0.49 a
	MR	27.51 ± 1.02 a	28.24 ± 0.73 a	28.57 ± 0.81 a	27.64 ± 0.97 a	27.70 ± 0.68 a	28.15 ± 0.87 a	28.10 ± 0.86 a
	CR	30.70 ± 0.97 a	29.56 ± 0.51 a	29.61 ± 0.24 a	29.58 ± 0.75 a	29.91 ± 0.55 a	29.06 ± 0.45 a	30.05 ± 0.55 a
RS	FR	27.24 ± 0.92 b	37.50 ± 1.14 a	25.39 ± 0.54 b	27.30 ± 1.15 b	36.11 ± 3.34 a	36.29 ± 0.76 a	36.02 ± 1.14 a
	MR	31.32 ± 0.58 c	39.91 ± 0.95 a	39.03 ± 1.43 ab	35.16 ± 1.20 abc	36.16 ± 3.58 abc	33.76 ± 2.10 bc	33.77 ± 1.55 bc
	CR	41.44 ± 1.48 a	22.60 ± 1.26 c	23.82 ± 3.45 bc	37.54 ± 1.03 a	27.73 ± 1.95 bc	29.96 ± 2.18 b	30.21 ± 2.09 b
RV	FR	5.24 ± 0.65 c	13.78 ± 0.94 a	5.02 ± 0.42 c	5.25 ± 0.59 c	11.73 ± 1.85 ab	9.21 ± 1.38 b	9.14 ± 1.33 b
	MR	14.75 ± 1.99 c	22.47 ± 0.83 ab	19.28 ± 0.71 abc	19.27 ± 0.62 abc	17.13 ± 3.70 bc	20.58 ± 2.17 abc	24.67 ± 2.90 a
	CR	80.00 ± 2.60 a	63.74 ± 1.62 c	75.70 ± 0.94 ab	75.48 ± 1.08 ab	71.14 ± 3.28 bc	70.22 ± 3.09 bc	66.18 ± 4.11 c

**Table 3:** Principal component analysis results of phenotypic traits of *A*. *fruticosa* seedlings under different treatments.

Index	PC1	PC2	PC3	PC4	PC5	PC6	PC7
H	−0.118	−0.555	−0.595	−0.282	−0.334	−0.135	0.127
BDH	0.266	0.606	0.461	−0.051	0.464	0.153	−0.062
LL	−0.091	−0.668	−0.359	−0.028	−0.193	−0.068	−0.392
LW	−0.020	0.153	0.187	0.829	−0.049	−0.093	−0.146
LA	−0.199	0.054	0.025	0.870	−0.101	−0.126	0.086
BBDH	0.372	0.408	0.594	0.114	0.303	0.259	−0.266
NB	0.143	−0.025	0.325	−0.057	0.789	0.071	0.201
BA	−0.343	0.070	0.097	−0.082	0.557	0.152	−0.348
TRL	0.228	0.894	0.263	0.048	0.042	0.238	−0.008
FRL	0.070	0.937	0.200	−0.092	0.081	0.116	0.120
MRL	0.461	0.082	0.293	0.415	−0.110	0.455	−0.389
CRL	0.534	0.350	−0.203	−0.156	0.404	0.162	−0.017
TRS	0.255	0.815	0.198	0.303	0.074	0.090	−0.062
FRS	0.147	0.935	0.125	0.079	−0.064	0.018	0.011
MRS	0.164	0.641	0.220	0.407	0.036	−0.080	−0.324
CRS	0.031	0.061	−0.212	−0.051	0.080	0.163	0.820
TRV	0.179	0.336	0.055	−0.160	0.470	0.671	0.294
FRV	0.027	0.862	0.126	−0.251	0.042	0.260	0.008
MRV	0.056	0.352	0.173	−0.116	0.115	0.806	0.043
CRV	0.206	0.186	−0.004	−0.129	0.556	0.557	0.361
LFW	−0.247	−0.346	−0.483	0.499	0.036	0.323	−0.058
SFW	−0.047	−0.457	−0.766	−0.238	−0.101	−0.064	0.109
LDW	−0.279	−0.351	−0.573	0.457	−0.250	0.028	0.284
SDW	−0.061	−0.321	−0.794	−0.219	−0.129	−0.111	0.142
FRFW	0.779	0.213	0.496	−0.067	−0.068	0.142	0.165
MRFW	0.788	0.248	0.435	−0.075	0.020	0.177	0.083
CRFW	0.804	0.177	0.475	−0.068	−0.054	0.134	0.160
FRDW	0.926	0.181	0.170	−0.060	0.101	−0.025	−0.054
MRDW	0.892	0.260	0.113	−0.058	0.137	0.000	−0.177
CRDW	0.929	0.154	0.140	−0.046	0.100	−0.054	−0.093
TFW	0.858	−0.136	−0.027	0.018	−0.078	0.286	0.215
TDW	0.917	−0.013	−0.327	−0.023	0.000	−0.067	0.028
RSR	0.624	0.243	0.645	−0.175	0.158	0.038	−0.148
Eigenvalue	13.534	5.064	3.374	2.296	2.004	1.265	1.002
Contribution rate (%)	41.013	15.345	10.224	6.959	6.071	3.832	3.035
Accumulated contribution rate (%)	41.013	56.358	66.582	73.541	79.612	83.444	86.479

**Table 4:** Principal component scores and ranking of comprehensive evaluation for different treatments.

Treatment Concentration	PC1	PC2	PC3	PC4	PC5	PC6	PC7	Comprehensive Score	Rank Order
SD3	5.25	1.72	1.66	−0.75	−0.94	0.50	0.62	2.91	1
SD4	0.43	1.74	1.54	1.23	0.90	0.86	0.21	0.90	2
D3	1.44	1.26	−1.82	−1.28	1.99	0.39	−0.26	0.74	3
SD2	0.60	1.69	0.50	−1.31	−0.74	−0.42	0.34	0.48	4
S2	1.81	−1.18	−2.82	1.67	−1.31	−0.03	0.09	0.36	5
SD1	−3.15	−0.89	0.18	0.43	1.15	−0.98	0.58	−1.55	6
CK	−6.38	−2.54	0.50	0.10	−1.05	0.12	−0.35	−3.49	7

**Table 5:** Treatment combinations and application parameters.

Treatments	Plant Buffer Concentration	Application Rate (mL)	Number of Plants
CK	Clear water	200	5
S2	S_3307_ 200 mg·L^–1^	200	5
D3	PP_333_ 300 mg·L^–1^	200	5
SD1	S_3307_ 100 mg·L^–1^ + PP_333_ 200 mg·L^–1^	200	5
SD2	S_3307_ 100 mg·L^–1^ + PP_333_ 300 mg·L^–1^	200	5
SD3	S_3307_ 200 mg·L^–1^ + PP_333_ 200 mg·L^–1^	200	5
SD4	S_3307_ 200 mg·L^–1^ + PP_333_ 300 mg·L^–1^	200	5

## Data Availability

The data presented in this study are available upon request from the corresponding author. The data are not publicly available due to ethical reasons.
